# Fiber Concentrate from Orange (*Citrus sinensis* L.) Bagase: Characterization and Application as Bakery Product Ingredient

**DOI:** 10.3390/ijms12042174

**Published:** 2011-03-29

**Authors:** Maria R. Romero-Lopez, Perla Osorio-Diaz, Luis A. Bello-Perez, Juscelino Tovar, Aurea Bernardino-Nicanor

**Affiliations:** 1 Centro de Desarrollo de Productos Bióticos del IPN, Km 8.5 carr. Yautepec-Jojutla, Colonia San Isidro, Apartado postal 24, 62731 Yautepec, Morelos, Mexico; E-Mails: shasen_33@yahoo.com (M.R.R.-L.); labellop@ipn.mx (L.A.B.-P.); 2 Instituto de Biología Experimental, Facultad de Ciencias, Universidad Central de Venezuela, Caracas, Venezuela and Functional Food Science Centre, Lund University. P.O. Box 124, 221 00 Lund, Sweden; E-Mail: drtovar@yahoo.com; 3 Departamento de Ingeniería Bioquímica, Instituto Tecnológico de Celaya, Av. Tecnológico y A. García Cubas S/N, Apartado postal 57, 38010 Celaya, Guanajuato, Mexico; E-Mail: aureabernardino@yahoo.com

**Keywords:** dietary fiber, orange, indigestible fraction, starch digestibility, muffin

## Abstract

Orange is a tropical fruit used in the juice industry, yielding important quantities of by products. The objective of this work was to obtain a dietary fiber-rich orange bagasse product (DFROBP), evaluate its chemical composition and its use in the preparation of a bakery product (muffin). Muffins containing two different levels of DFROBP were studied regarding chemical composition, *in vitro* starch digestibility, predicted glyceamic index and acceptability in a sensory test. DFROBP showed low fat and high dietary fiber contents. The soluble and insoluble dietary fiber fractions were balanced, which is of importance for the health beneficial effects of fiber sources. DFROBP-containing muffins showed the same rapidly digestible starch content as the reference muffin, whilst the slowly digestible starch level increased with the addition of DFROBP. However, the resistant starch content decreased when DFROBP increased in the muffin. The addition of DFROBP to muffin decreased the predicted glyceamic index, but no difference was found between the muffins prepared with the two DFROBP levels. The sensory score did not show difference between control muffin and that added with 10% of DFROBP. The addition of DFROBP to bakery products can be an alternative for people requiring low glyceamic response.

## 1. Introduction

Obesity is an important health problem in Mexico and worldwide. Among current dietary trends, consumption of food products with reduced content of digestible carbohydrates has gained in popularity. Dietary fiber (DF) is a food ingredient that is neither digestible nor absorbed in the small intestine of the human. The development of new products with substantial DF contents is a strategic area for the food industry. Consumers are demanding foods that show two main properties: the first one refers to the traditional nutritional aspects of the food, whereas, as a second feature, additional health benefits are expected from its regular ingestion. Foods complying with these requisites are often called functional or nutraceutical foods.

In a rapidly changing world, with altered food habits and stressful life styles, it is more and more recognized that a healthy digestive system is essential for the overall quality of life [1]. DF plays an important role in decreasing the risks of many disorders such as intestinal constipation, diabetes, cardiovascular diseases, diverticulosis and obesity [2]. Also, DF may reduce insulin secretion by slowing the rate of nutrient absorption following a meal, a property that is particularly associated to the soluble fraction of fiber. Experimentally, insulin sensitivity tends to increase and body weight decreases on high-fiber diets [3].

Most fractions (cellulose, lignin, hemicellulose, pectins, gums and mucilages) of DF are the major constituents of plant cell walls [4]. Although some authors recommend dropping the terms “soluble” and “insoluble” fiber, the physiological effects of this indigestible component of foods is being increasingly recognized [5].

Among good sources of fiber cereal grains, legumes, fruits (tropical), vegetables, nuts and seeds are of importance. These sources include citrus, as it exhibits 25–70% fiber content [6]. Approximately 50% of the orange fruit is juice, while the other 50% is the rind, albedo, sacs and seeds, which contain varying amount of fiber [7]. The fruits and their by-products can be dried for preservation and further use, which enables the exploit of features of interest, *i.e.*, low in fat and digestible carbohydrates, high in fiber and low calorie content [8]. Thus, one important source of citrus dietary fiber is the residue from the orange juice industry. Fiber from citrus can be obtained from edible parts [9,10] and attracts, binds, and manages high levels of water (up to 12 times its weight) in baked goods, meat and poultry products, and sauces. Additionally, citrus peel is a rich source of fiber and antioxidant, but the high levels of astringent compounds make it unsuitable for human consumption [11]; however, there is a commercial product, CitraFiber^™^ by Natural Citrus Products (LaBelle, FL) that is used in bakery products.

Fibers traditionally used for food processing are derived from cereals. Diverse studies have been conducted to obtain and assess the composition of dietary fiber-rich products obtained from by-products of diverse vegetable sources such as passion fruit, apples, pears, oranges, peaches, artichokes, asparagus, lemon, black currant, pear, cherry and carrot [12–14].

Starch is the major digestible carbohydrate in human diet [15], representing most of the “available” or “glycemic” carbohydrates, defined as those digested by human digestive enzymes in the gastrointestinal tract and absorbed into the bloodstream as glucose [16]. However, carbohydrates that restrict access of digestive enzymes to the starch substrate, such as certain dietary fiber types, produce a slow release of glucose from the food matrix, prolonging the digestion process [17].

The rate at which starch and other carbohydrates are digested and absorbed in the small intestine, has received great interest because of its association with the glycemic response and postprandial metabolism. Most baked goods contain free sugars and gelatinized starch, which have a readily dispersible in the food matrix. Starch digestibility can be affected *in vitro* and *in vivo* by the macro-food properties (e.g., plant tissues containing intracellular starch granules and the starch-gluten matrix in white bread), the presence of other dietary compounds as fiber and lipids, as well as anti-nutrients (protein inhibitors of α-amylase; polyphenols). Also the structure and physicochemical properties of native (raw) starch granules (granule size, amylose-amylopectin ratio and type of crystallinity) may influence the kinetics and extent of the polymer digestion [18].

It has been observed that co-ingestion of starch and soluble fibers results in slowered gastric emptying, which may also contribute to reduced postprandial blood glucose and insulin levels and thus influence satiety [19].

Since and increased intake of DF is generally believed to be an effective way for prevention of chronic diseases, this ingredient is used in a variety of foods such as bars, cookies, soups, beverages, confectionery, snacks, in which has. Over the years, various fibers sources have fallen into and out of acceptance by the food industry and consumers alike. However, the use of fiber foods has continued to grow and expand, with ever-incrementing numbers of available applications [6].

In view of the nutritional and technological relevance of dietary fiber, and the considerable volume of sweet orange bagasse disposal by the juice industry, the objective of this study was to obtain and characterize a dietary fiber-rich product from orange bagasse. The product was used to elaborate composite muffins whose chemical composition, starch digestibility, predicted glycemic index and sensory characteristics were assessed.

## 2. Results and Discussion

### 2.1. Chemical Composition

Dietary fiber-rich orange bagasse product (DFROBP) exhibited low moisture content (Table 1), similar to those determined in dry by-products of orange (6.0 g/100 g dry sample) [8]. An important parameter of any DF ingredient such as DFROBP is its fat content. The recorded value (0.6 g/100 g dry sample) is lower than those reported in citrus peels such as orange (1.64 g/100 g dry sample), grapefruit (2.01 g/100 g dry sample) and mandarin (1.45 g/100 g dry sample) [20]. Fruits are characterized by their content of different minerals; DFROBP showed a 2.6 g/100 g dry sample ash content, which is similar to that determined in grapefruit peel with 2.99 g/100 g dry sample [20] and in orange by-products with 2.5 g/100 g dry sample [8], but lower than in mandarin peel (3.96 g/100 g dry sample; [20]) and lemon (3.91 g/100 g dry sample; [21]. Compared with other fiber ingredients, such as lemon peel (6.79 g/100 g dry sample) and grapefruit (8.42 g/100 g dry sample) [21], the protein content of DFROBP was relatively low (4 g/100 g dry sample) However, it was similar to those reported for fibers derived from other by-products of the orange-processing industry (6.0 g/100 g dry sample) [12].

It is important to highlight the low total starch content of DFROBP (7.1 g/100 g dry sample) (Table 1). Such a value is markedly smaller than in other fruit-derived dietary fiber products, like a mango dietary fiber preparation (29.88 g/100 g dry sample; [22]. Total dietary fiber (TDF) content in DFROBP was high (41.5 g/100 g dry sample; Table 1), a value that resembles that found in grapefruit peel (44.2 g/100 g dry sample) and orange peel (49.78 g/100 g dry sample) [20,21], but higher than in mango dietary fiber (28.05 g/100 g dry sample) [22]. The relative content of soluble (SDF) and insoluble (IDF) dietary fiber fractions is considered relevant from a nutritional and functional point of view. DFROBP shows a good balance of both components since similar contents were recorded for both fractions. SDF content in DFROBP was higher than in unripe banana flour (5.44 g/100 g dry sample) and apple (5.05 g/100 g dry sample) [23]. The IDF content in DFRBP, on the other hand, was higher than in mango dietary fiber (13.80 g/100 g dry sample) [22], but lower than in grapefruit peels (46.44 g/100 g dry sample) and orange (48.03 g/100 g dry sample) [20]. A similar pattern was obtained for the total indigestible fraction (TIF), which was higher compared with unripe banana (36.08 g/100 g dry sample) and apple (16.97 g/100 g dry sample) [24].

### 2.2. Chemical Composition of Bakery Products

Moisture content was similar in the two muffins containing DFROBP and lower than in the control product (Table 2), showing that final moisture was affected by the inclusion of DFRBP in the formulation. Development of food products with low moisture content is important to achieve increased shelf-live [25].

No differences between control and DFROBP containing samples were found regarding fat and protein contents (Table 2). The lower protein level is probably due to the reduced contribution of proteins from the wheat flour in the composite muffins. Ash content differed between control and experimental muffins reflecting DFROBP mineral contribution. Both products formulated with DFROBP exhibited increased TDF levels (Table 2), with a higher content of IDF. Addition of DFROBP also increased significantly (α = 0.05) the SDF content of muffins, from a control value of 1.8 g/100 g dry sample to 2.8–3.0 g/100 g dry matter in the composite samples. TDF content in the muffins containing DFROBP was similar to that found in bread prepared with mango dietary fiber (16.6 g/100 g dry sample) [19] and higher than in breads containing chia (*Salvia hispanica* L.) (2.25 g/100 g dry sample) or flaxseed (*Linum usitatissimum* L.) (1.41 g/100 g dry sample) [26].

As in the case of DF, the total indigestible fraction (TIF) content of muffins increased with the addition of DFROBP (Table 2). TIF levels were higher than in white bread (11.06 g /100 g dry sample) and refined flour-based biscuits (10.28 g/100 g dry sample) [24]. Soluble indigestible fraction (SIF) in muffin with 15% of DFROBP (8.8 g/100 g dry sample) was higher than white bread (2.78 g /100 g dry sample) and refined flour-based biscuits (2.65 g/100 g dry sample) [24]. Similarly, the insoluble indigestible fraction (IIF) content of the muffin prepared with 15% of DFROBP (27.4 g/100 g dry sample) was notably higher than in white bread and biscuits studied by Saura-Calixto & Goñi [24]. Thus, indigestible fraction values corroborate that there is an increase in non-digestible components after incorporation of DFROP in the muffin formulation, a fact that is considered of physiological importance in view of their potential as substrate for the colonic flora [27].

### 2.3. *In Vitro* Starch Digestibility

The muffins elaborated with DFROBP had similar total starch contents (Table 3). This observation may be explained by the relatively low levels of DFROP incorporated in the blends; thus the small dilution effect caused by the ingredient was not detected in terms of starch content of the final composite baked product. Other bakery products, such as cookies elaborated with mango dietary fiber ingredient [22] or with banana resistant starch-rich powder [28], show lower total starch content (45.5% and 48.5%, respectively). Most commercial cereal products, for instance cornflakes and others, have higher total starch contents [17].

Studies in humans have demonstrated that incorporation of slowly digestible starch (SDS) and resistant starch (RS) in the diet can produce health benefits [29]. Approximately 60% of the starch present in the muffins was rapidly digestible starch (RDS) (Table 3). Cooked/processed cereals are characterized by high slowly digestible starch (SDS) contents. Boiled maize starch, for example, contains 85% RDS [30]. Diverse commercial cereal products and crackers have RDS contents ranging between 58 and 79% [17]. In the present work, the highest SDS content was recorded in the muffin prepared with 15% of DFROBP and the lowest one in control muffin. Furthermore, the SDS content in the 15 DFROP muffin almost doubled that recorded in the reference muffin (Table 3). Formulations containing significant levels of dietary fiber, as here-studied muffins, may exhibit significant viscosity [31], which may result in decreased hydrolysis rate of the starch present in the baked product. Different commercial cereal products have lower SDS (3.0%) [17] than those determined in the studied muffins, suggesting the nutraceutical potential of our bakery product containing DFROBP, which can place it as an alternative item for special dietary regimes. Consumption of high SDS-products is considered beneficial, as they should not produce the postprandial hyperglycemic and hyperinsulinemic spikes associated with RDS-rich meals [32].

RS contents followed an inverse pattern to that observed for SDS. The values recorded in the DFROP-containing muffins were lower than those estimated in the reference product (Table 3). This may be considered an indicative of reduced formation of indigestible retrograded starch as consequence of the augmented DF content in the baked product. Although RS determined in some commercial products as Special K^®^ (1.56%), and different crackers (1.6–1.7%) [17] are lower than those recorder here for the experimental muffins, the RS-increasing power of DFROP is far below that of a banana resistant starch-rich powder described in the literature, which allowed the production of prototype composite cookies with a 8.42% RS value [28].

### 2.4. *In Vitro* Kinetic of Starch Digestion

**Figure 1.**
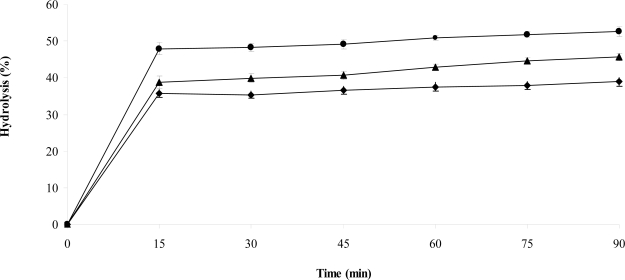
Hydrolysis rate of muffin prepared with DFROBP at different level. • Control muffin; ▴ Muffin 10%; ♦ Muffin 15%.

Percentage of starch hydrolyzed at 90 min (H_90_) (Figure 1) and the corresponding predicted glycemic indexes (pGI) are presented in Table 4. Although the three muffins exhibited low digestion rates, the control muffin had the highest H_90_ value, while the muffins with DFROBP decrease the hydrolysis rate of starch. H_90_ and GI are influenced by physical characteristics of the food products such as texture (hardness, porosity), particle size and viscosity, as well as by intrinsic characteristics such as structure and physicochemical properties of the starch substrate. In this sense, it is more meaningful to assess H_90_ as a physiologically closer parameter than the simple RDS/SDS content.

Soluble components of dietary fiber (Tables 1 and 2) can slow not only digestion but also diffusion of digestion products to the absorptive mucosa [33,34]. Perhaps the higher soluble indigestible fraction of muffins prepared with DFRBP (Table 2) increases the viscosity and retards the absorption phase of the digestion, resulting in a rather “slow” feature.

The pIG suggests important “slow digestion” features for the experimental muffins. A decrease of 10 points in pGI was recorded in muffins with DFROBP compared with the control muffin. Higher GI values for cornflakes (93) and Special K^®^ (84) have been determined, while commercial cereal-based crackers had GI values ranging between 52 and 64 [17]. It would be worthwhile confirming the low *in vivo* GI of muffins prepared with DFROBP as this characteristic may be useful for the dietary management of people with impaired glucose tolerance.

### 2.5. Preference Test

Table 5 presents the acceptability of muffins using a hedonic scale. Control muffin and that with 10% DFROBP were similarly accepted, whereas a lower acceptability score was registered for muffin with the highest DFROBP level. Since the chemical composition, starch digestibility and pGI characteristics of muffins with DFRBP are similar, the sensorial acceptability may represent a criterion to select the most appropriate formulation for future applications.

## 3. Experimental Section

### 3.1. Materials

Sweet oranges (*Citrus sinensis*) from Ixthuatlán, Veracruz, Mexico were used. The peel of the orange was removed with an orange peeler and juice was extracted manually. The bagasse left was dried at 60 °C for 8 h in a tray dryer. The dry bagasse was ground in a manual mill (Del rey, Mexico) and sieved through a mesh number 40 (425 μm).

### 3.2. Preparation of Muffins

The muffin ingredients (margarine, egg, baking powder and sugar) were acquired in the local market. Wheat flour was provided by Selecta, S.A. de C.V. Mexico. The formulations of these muffins are shown in Table 6, with wheat flour as the basis for control muffin and two wheat flour/orange bagasse blends (10 and 15% orange bagasse) as main ingredient of the experimental muffins. Margarine (containing a blend of vegetable oils, whey milk, soy lecithin and citric acid) was creamed, mixed with confectioner’s sugar and a whole egg, added to the wheat flour or the wheat flour/orange bagasse blend and mixed thoroughly. The muffins were baked in a household oven, at an approximate temperature of 180 °C for 45 min. Once baked, muffins were allowed to cool down to room temperature for 45 min and stored in a plastic container with hermetic cover.

### 3.3. Chemical Composition

Moisture content was determined by heating (110 °C for 3 h) using 2 g of sample. Ash, protein and fat were analyzed according to AACC methods 08-14, 42-11, and 32-25, respectively [35]. Total starch was determined by an enzymatic/colorimetric method [36]. The total dietary fiber (TDF) content was determined with the 32-05 AACC method [35]. Soluble (SIF) and insoluble (IIF) indigestible fractions were assessed using the sequential pepsin/amylase hydrolysis protocol of Saura-Calixto *et al.* [23].

### 3.4. Total, Rapidly Digestible, Slowly Digestible and Resistant Starch Fraction

The rapidly, slowly digestible and resistant starch fractions were determined with the procedure proposed by Englyst, Kingman & Cummings [37].

### 3.5. *In Vitro* Kinetic of Starch Digestion

The *in vitro* rate of hydrolysis was measured using hog pancreatic α-amylase according to Holm *et al.* [38] with minor modifications. A 50 mL of phosphate buffer (pH 6.9) were added to a portion of each sample containing 500 mg of starch. Samples were incubated a 37 °C in a shaking water bath. In the first 5 min before the addition of enzyme aliquots of 0.2 mL of each sample were taken to mark as time zero. After an interval of 1 min, 1 mL of a solution containing 40 mg of porcine pancreatic α-amylase (A-3176, Sigma Chemical Co.) in 1 mL of phosphate buffer was added to each sample. Samples (0.2 mL) were withdrawn after 15 min and every 15 min for 90 min. These samples were added to tubes than containing 0.8 mL distilled water and 1 mL of 3,5 dinitrosalicylic acid (DNS). Samples were incubated at 100 °C in water bath for 10 min. Then 15 mL of distilled water was added to each tube and mixed well. The reducing sugars released were measured at 530 nm in parallel with a standard curve of maltose. The rate of hydrolysis was expressed as the percentage of starch hydrolyzed with respect to dry matter at different times.

The predicted glycemic index (pGI) was calculated from percentage of starch hydrolyzed at 90 min (H_90_) values using the formula proposed by Goñi *et al.* [36]: pGI = 39.21 + 0.803 (H_90_) (*r* = 0.909, *p* ≤ 0.05).

### 3.6. Sensory Analysis of Muffins

This test was applied to muffins prepared with two levels of bagasse flour (0, 10 y 15%). Participants were untrained judges chosen at random from personnel at Centro de Desarrollo de Productos Bióticos, using a preference scale (Table 7). A total of 100 volunteers (61 women and 39 men) between 18 and 58 years old took part in the survey. The stimuli were placed on separate plastic trays and labeled with three digit random numbers. The order of presentation of the stimuli was counterbalanced over consumers. Each consumer tasted approximately 1 g of each sample. Rinses were taken before tasting and swallowing the samples.

### 3.7. Statistical Analysis

Results were expressed as means of values ± standard error of the separate determinations. Comparison of means was performed by one-way analysis of variance (ANOVA) followed by Tukey’s test.

## 4. Conclusions

A dietary fiber-rich orange bagasse product (DFROBP) with a total dietary fiber (TDF) content of 41.5% was prepared. The product increased the fiber content of experimental muffins by 40 and 63% compared to a control muffin. Although no difference in the rapidly digestible starch level was found between control muffin and those containing DFROBP, increased slowly digestible starch contents were recorded after the addition of this ingredient. However, resistant starch levels decreased with the addition of DFROBP. DFROBP-added muffins showed an importantly decreased predicted glycemic index, and the preference test indicated similar acceptability to the control and the 10% substituted muffin. Partial wheat flour substitution with DFROBP allowed production of prototype baked products containing high levels of TDF and indigestible fraction, features that may be of use in dietary regimes for people with different nutritional requirements.

## Figures and Tables

**Table 1.** Chemical composition of dietary fiber-rich orange bagasse products.

**Components**	**Amount (g/100g)**
Moisture	9.9 ± 0.0
Ash	2.6 ± 0.0
Protein	4.0 ± 0.0
Lipids	0.6 ± 0.5
Total dietary fiber	41.5 ± 0.0
Soluble Dietary fiber	18.6 ± 0.8
Insoluble dietary fiber	22.9 ± 0.6
Total starch	7.1 ± 0.4
Total indigestible fraction	59.1 ± 0.2
Soluble indigestible fraction	20.2 ± 0.2
Insoluble indigestible fraction	38.9 ± 0.2

* Average of three replicates ± standard error

**Table 2.** Chemical composition of muffins with two levels of DFROBP and control muffin.

**Components**	**Samples**		
	**Control Muffin**	**Muffin 10%**	**Muffin 15%**
Moisture	35.2 ± 0.14^a^	28.4 ± 0.45^b^	27.9 ± 0.28^b^
Ash	2.2 ± 0.01^a^	2.5 ± 0.02^a^	3.7 ± 0.01^c^
Proteins	9.7 ± 0.03^a^	9.1 ± 0.03^a^	8.9 ± 0.11^a^
Lipids	15.5 ± 0.03^a^	15.5 ± 0.11^a^	15.3 ± 0.35^a^
Total dietary fiber	9.2 ± 0.21^a^	12.9 ± 0.40^b^	15.0 ± 0.35^c^
Soluble dietary fiber	1.8 ± 0.06^a^	2.8 ± 0.12^b^	3.0 ± 0.02^b^
Insoluble dietary fiber	7.4 ± 0.06^a^	10.1 ± 0.12^b^	12.0 ± 0.02^c^
Indigestible fraction	20.0 ± 0.22^a^	25.2 ± 0.31^b^	27.4 ± 0.27^c^
Soluble indigestible fraction	5.7 ± 0.09^a^	7.6 ± 0.26^b^	8.8 ± 0.26^c^
Insoluble indigestible fraction	14.3 ± 0.32^a^	17.6 ± 0.26^b^	18.6 ± 0.24^c^

a Average of three replicates ± standard error

b Different lowercase letters in the same row indicate significant difference (α = 0.05)

c DFROBP= dietary fiber-rich orange bagasse product

**Table 3.** Rapidly digestible (RDS), slowly digestible (SDS), resistant starch fractions (RS), and total starch (TS) in muffins with DFROBP and control muffin.

**Fractions**	**Control Muffin**	**Muffin 10%**	**Muffin 15%**
RDS	61.0 ± 0.6^a^	60.9 ± 0.6^a^	59.1 ± 0.6^a^
SDS	4.5 ± 1.0^a^	5.4 ± 0.9^b^	8.6 ± 0.8^c^
RS	9.5 ± 0.5^a^	3.9 ± 0.3^b^	2.0 ± 0.3^c^
TS	75.0 ± 0.7^a^	70.2 ± 0.8^b^	69.6 ± 0.6^b^

a Average of 100 replicates ± standard error

b Different lowercase letters in the same row indicate significant difference (α = 0.05)

c DFROBP = dietary fiber-rich orange bagasse product

**Table 4.** Starch hydrolyzed at 90 min and predicted glycemic index.

	**Control Muffin**	**Muffin 10%**	**Muffin 15%**
H_90_ (%)	52.7 ± 0.3^a^	45.6 ± 0.3^b^	38.9 ± 0.4^c^
pIG^1^	81.5^a^	75.8^b^	70.4^c^

a Values are mean ± SEM, *n* = 3, dry matter

b Means with different letters in rows are significantly different (α = 0.05)

1 Prediction of glycemic index (pGI) = 39.21 + 0.803 (H_90_) (Goñi *et al.*, 1997)

c DFROBP = dietary fiber-rich orange bagasse product

**Table 5.** Sensory analysis of muffins with DFROBP and control muffin.

**Samples**	**Qualification**
Control muffin	6.3 ± 0.1^a^
Muffin 10%	6.0 ± 0.2^a^
Muffin 15%	4.2 ± 0.2^b^

a Average of 100 replicates ± standard error

b Different lowercase letters in the same column indicate significant difference (α = 0.05)

c DFROBP = dietary fiber-rich orange bagasse product

**Table 6.** Formulation of control and composite muffins containing two different DFROBP levels.

**Ingredients (g)**	**Control**	**10%**	**15%**
Wheat flour	100	90	85
Bagasse	-	10	15
Sugar	37	37	37
Baking powder	4	4	4
Egg	1	1	1
Butter	27	27	27
Milk (mL)	109	109	109

DFROBP = Dietary fiber-rich orange bagasse product

**Table 7.** Nine-Point hedonic scale used in the preference test, with the corresponding Spanish translation.

**English**	**Spanish**
Like extremely	Gusta muchísimo
Like very much	Gusta mucho
Like moderately	Gusta moderadamente
Like slightly	Gusta poco
Neither like nor dislike	Ni gusta ni disgusta
Dislike slightly	Disgusta poco
Dislike moderately	Disgusta moderadamente
Dislike very much	Disgusta mucho
Dislike extremely	Disgusta muchísimo

Hedonic scale: 1 = dislike extremely, 5 = neither like nor dislike, 9 = like extremely
